# Supplementary material for: A Novel Thienopyrimidine Analog, TPH104, Mediates Immunogenic Cell Death in Triple-Negative Breast Cancer Cells
Source: Cancers (Basel). 2021 Apr 18;13(8):1954. doi: 10.3390/cancers13081954 (PMC8074041; doi:10.3390/cancers13081954)
Supplement: Supplementary file 1 [file cancers-13-01954-s001.zip › cancers-1158159-supplementary.pdf]

cancers

MDPI

Article:

Supplementary File:

A Novel Thienopyrimidine Analog, TPH104, Mediates Immunogenic Cell Death in Triple-Negative Breast Cancer Cells

Diwakar Bastihalli Tukaramrao [1,†], Saloni Malla [1,†], Siddharth Saraiya [2], Ross Allen Hanely [1], Aniruddha Ray [3], Shikha Kumari [1], Dayanidhi Raman [4] and Amit K. Tiwari [1,*]

[1] Department of Pharmacology and Experimental Therapeutics, College of Pharmacy & Pharmaceutical Sciences, University of Toledo, Toledo, OH 43614, USA; diwakarbt02@gmail.com (D.B.T), saloni.malla@rockets.utoledo.edu (S.M.), ross.hanely@utoledo.edu (R.A.H.), shikha.pharma08@gmail.com (S.K.)
[2] Department of Radiation Oncology, College of Medicine, University of Toledo, Toledo, OH 43614, USA; saraiya.s@gmail.com
[3] Department of Physics, College of Natural Sciences, University of Toledo, Toledo, OH 43614, USA; aniruddha.ray@utoledo.edu
[4] Department of Cancer Biology, College of Medicine, University of Toledo, Toledo, OH 43614, USA; dayanidhi.raman@utoledo.edu
* Correspondence: amit.tiwari@utoledo.edu; Tel.: (+14193831913)
† Equal contributions

Citation: Bastihalli Tukaramarao, D.; Malla, S.; Saraiya, S.; Hanely, R.; Ray, A.; Kumari, S.; Raman, D.; Tiwari, A. A novel thienopyrimidine analog, TPH104, mediates immunogenic cell death in triple negative breast cancer cells. Cancers 2021, 13, x. https://doi.org/10.3390/xxxxx

Academic Editor: Sara A. Hurvitz

Received: 9 March 2021
Accepted: 13 April 2021
Published: date

Publisher's Note: MDPI stays neutral with regard to jurisdictional claims in published maps and institutional affiliations.

Copyright: © 2021 by the authors. Submitted for possible open access publication under the terms and conditions of the Creative Commons Attribution (CC BY) license (http://creativecommons.org/licenses/by/4.0/).

Supplementary Figure S1

Figure S1. (A) Schematic representation of the protocol employed for the collection of conditioned media and ex vivo culture and stimulation of BMDCs. (B) Percentage of triple positive (CD11C+, MHCII+, CD86+) BMDCs after incubation for 24h in culture media spiked with conditioned media (CM) in the ratio of 7.5:1. The conditioned media were collected from cultures of MDA-MB-231 cells after 24, 48 and 72h of treatment with either vehicle (VC-CM), TPH104; 1μM (TPH104-1-CM), TPH104; 5μM(TPH104-5-CM) or Mitoxantrone; 1μM (MX-1-CM). For UT-C complete media was used in place of conditioned media. The optimal BMDC stimulatory effect was observed induced by CM collected at 48h post treatment. Flow cytometry acquisition and analysis was performed on BD Accuri C6 plus Flow Cytometer.

Supplementary Figure S2

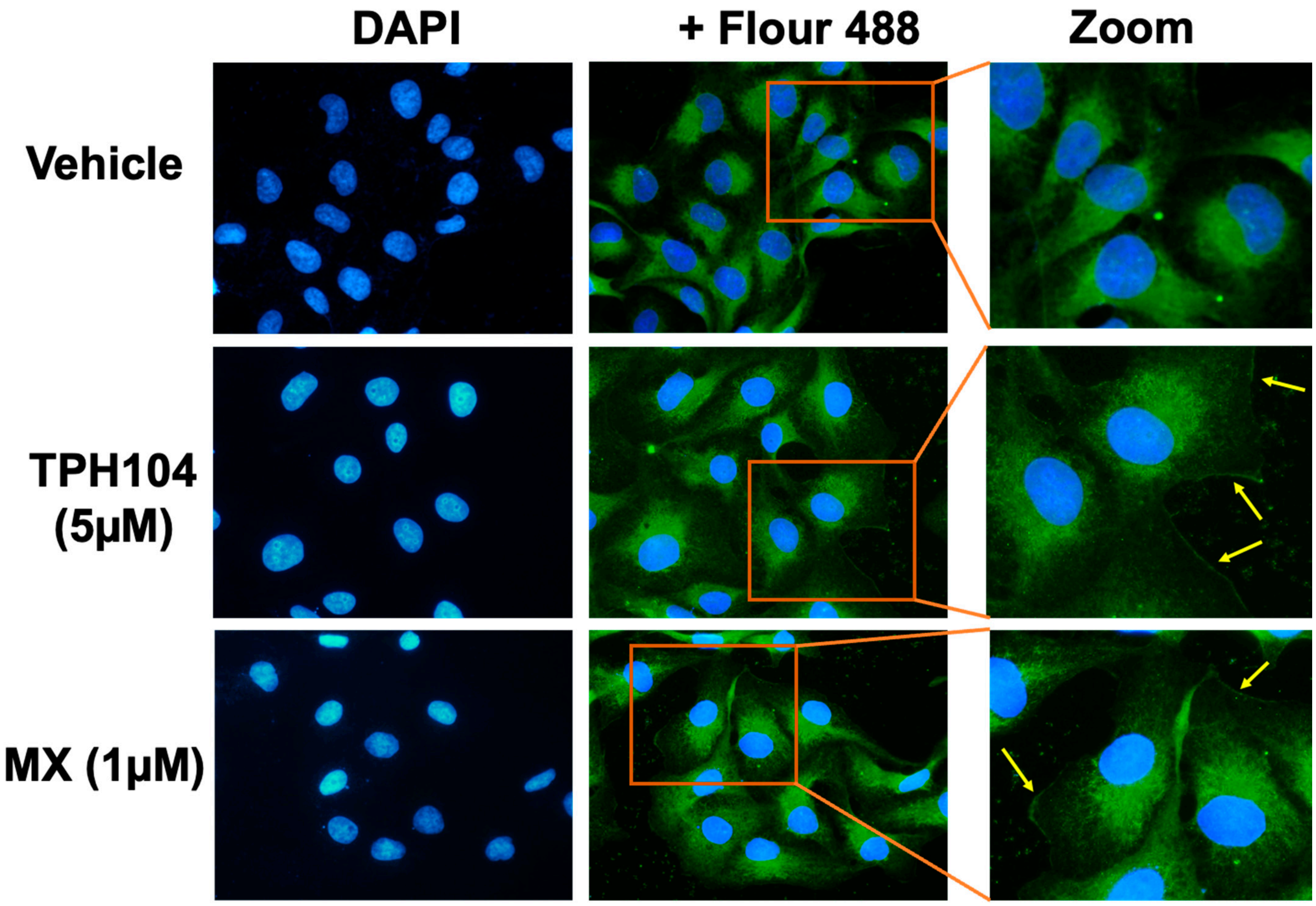

Figure S2. CRT translocate to the cell surface upon incubation with TPH104. Immunofluorescence detection of CRT (appearing in green, FITC) in the intracellular space and on the on the cell surface after treatment of MDA-MB-231 cells with either TPH104 (5μM; 6h), and mitoxantrone (MX; 1μM; 2h). The nuclei were visualized with DAPI. Yellow arrows indicate concentration of CRT in the plasma membrane microdomains in cells incubated with either TPH104 or MX.

Supplementary Video S1

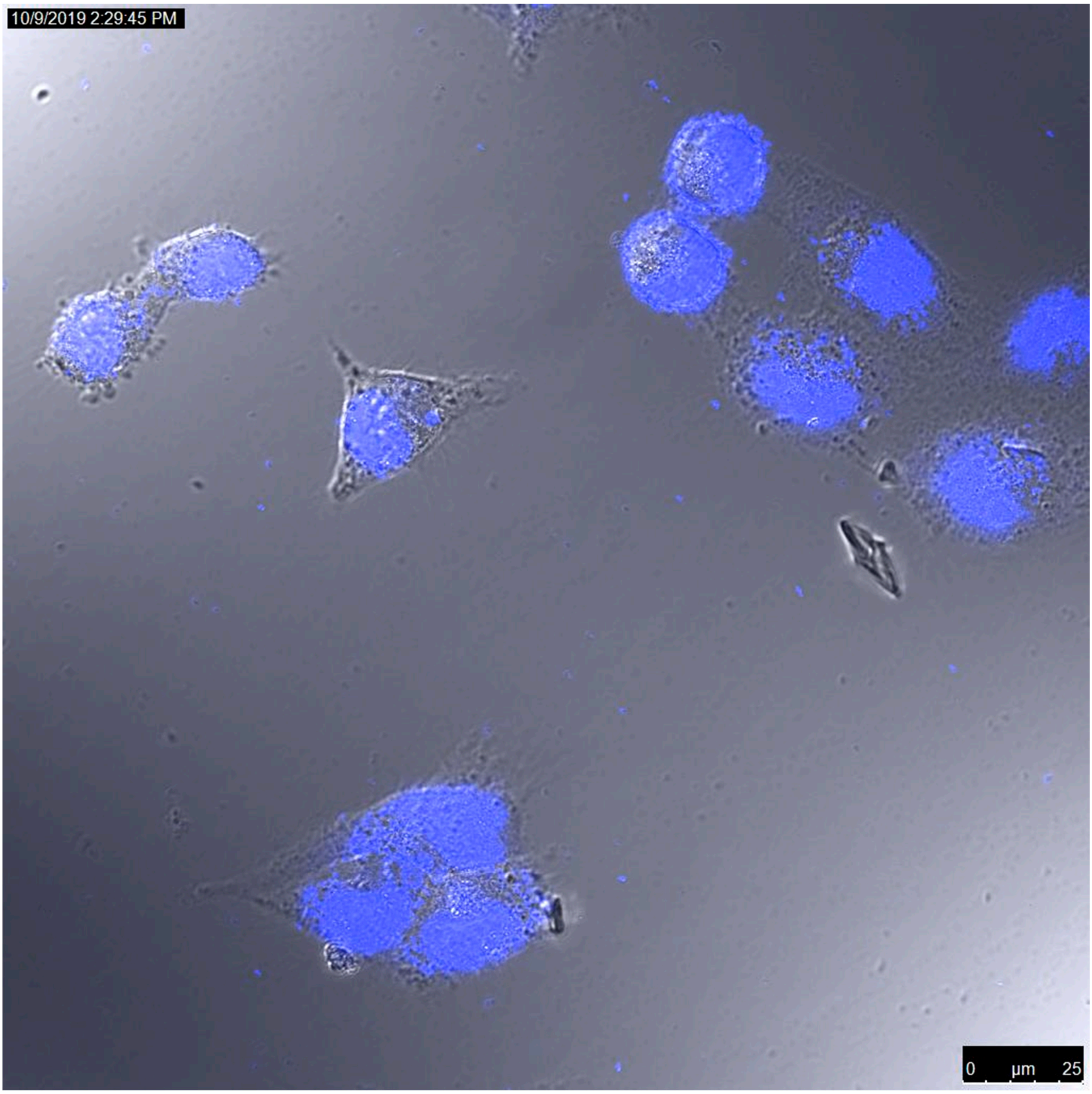

Video S1. Time-lapse movie, bright field merged with DAPI blue fluorescence shows the Morphology of MDA-MB-231cells during first 30 mins of incubation with TPH104 (10µM).
